# Toward Exploring the Structure of Monolayer to Few-layer TaS_2_ by Efficient Ultrasound-free Exfoliation

**DOI:** 10.1186/s11671-018-2439-z

**Published:** 2018-01-15

**Authors:** Yiwei Hu, Qiaoyan Hao, Baichuan Zhu, Biao Li, Zhan Gao, Yan Wang, Kaibin Tang

**Affiliations:** Department of Chemistry, Division of Nanomaterials and Chemistry, Hefei National Laboratory for Physical Sciences at the Microscale, University of Science and Technology of China, Hefei, 230026 People’s Republic of China

**Keywords:** Tantalum disulfide, Layer material, Exfoliation, Ultrasound-free, Structural transition

## Abstract

Tantalum disulfide nanosheets have attracted great interest due to its electronic properties and device applications. Traditional solution-ased ultrasonic process is limited by ultrasound which may cause the disintegration into submicron-sized flake. Here, an efficient multi-step intercalation and ultrasound-free process has been successfully used to exfoliate 1T-TaS_2_. The obtained TaS_2_ nanosheets reveal an average thickness of 3 nm and several micrometers in size. The formation of few-layer TaS_2_ nanosheets as well as monolayer TaS_2_ sheets is further confirmed by atomic force microscopy images. The few-layer TaS_2_ nanosheets remain the 1T structure, whereas monolayer TaS_2_ sheets show lattice distortion and may adopt the 1H-like structure with trigonal prism coordination.

**Electronic supplementary material:**

The online version of this article (10.1186/s11671-018-2439-z) contains supplementary material, which is available to authorized users.

## Background

Layered transition metal dichalcogenides (TMDs) have attracted great interest as two-dimensional materials due to their unique structural features and outstanding performances in the field of energy [1–9] and electricity [10–14]. Because of their lamellar structures, TMDs usually serve as the hosts for the intercalation of a wide variety of electron-donating species ranging from Lewis bases to alkali metals and can be exfoliated into nanosheets using organolithium [14] or alkali metal naphthalene [15] reduction chemistry and ultrasound physics [5, 9, 11, 15–19]. The exfoliated TMDs usually show enhanced properties in the field of electric [11, 14], catalyst [5, 6, 8, 9], and energy storage [2–4, 7].

As one of the typical TMD isomorphism, tantalum disulfide (TaS_2_) shows a unique combination of valuable structural, mechanical, chemical, and electronic properties, which has been studied for decades [10, 12, 15, 20–22]. Its lamellar structure and electronic properties are beneficial for high-power applications such as micro-electromechanical system, device applications [18, 21, 23], and superconductor [10, 12]. 1T and 2H phases are the most representative structures. The distinction between two phases can be the S-Ta-S coordinated situations, which are recognized as octahedral coordination (1T) and prismatic coordination (2H). The two phases are quite different in electronic structures and other properties. For example, bulk 1T-TaS_2_ shows charge density wave (CDW) transition and such a transition is found to vanish measured at (001) oriented single-crystal nanoflake (< 5 nm thickness) [18, 21, 23]. Whereas, the 2H phase shows superconductivity and its critical temperature can be raised by intercalation [24–26].

TMDs including TaS_2_ can be successfully exfoliated by mechanical method [22] or ultrasound-assisted intercalation process [1, 4–9, 14, 15, 19]. The exfoliated TaS_2_ obtained by mechanical method is only used to explore its microscopic properties [20, 23, 27]. All the other methods have not got rid of the physical ultrasound assistance [15–17, 19], which may suffer the size of the exfoliated nanoflakes [15] and limits their wide applications. Therefore, it is necessary for us to explore an efficient alternative exfoliation route.

Our search leads us to a multi-step intercalation method using alkali metal-ammonia as the first step intercalation reaction. In sodium liquid ammonia solution, for example, the metal transfers an electron to the solution to produce a radical solvated electron. The reaction introduces both alkali metal and ammonia molecule into the interlayer of TaS_2_. Moreover, the ammonia can be replaced by hydroxyl-contained water [28]. Hydroxyl-contained glucose was ever used to intercalate perovskite [29, 30] which can produce very large layer spacing. It is interesting to consider whether the hydroxyl-contained glucose can get into the TaS_2_ layer just like water and achieve the exfoliation of TaS_2_. And water-organic mixed solvent was ever employed to exfoliate high-quantity two-dimensional material [31, 32].

Herein, using a multi-step ultrasound-free intercalation process, we report efficient exfoliation of 1T-TaS_2_. The exfoliated TaS_2_ nanosheets show 1T structure with several micrometers in size and an average thickness of about 3 nm. Monolayer TaS_2_ can also be obtained, and some work was done to explore its structure features.

## Experimental

Ta, Na, and S power are available from Aladdin reagent. All reagents were used as received without further purification.

First, 1T-TaS_2_ was prepared by sealing a stoichiometric amount of Ta and S in vacuum quartz tube, then quenching the tube after heating to 900 °C for 1 day.

The exfoliation mechanism can be attributed to a multi-step intercalation process. Na_*x*_(NH_3_)_*y*_TaS_2_ was synthesized through liquid ammonia procedure. First, a sealed reaction bulb with certain amounts of sodium was placed under − 80 °C to obtain the ammonia solution of sodium. Then, the TaS_2_ powder was added into the deep-blue solution with a molar ratio of TaS_2_:Na being 1:0.8. After a 10–20-min reaction, liquid ammonia evaporated and Na_*x*_(NH_3_)_*y*_TaS_2_ was available.

Fifty milligrams of Na_*x*_(NH_3_)_*y*_TaS_2_ was exposed in air to transfer to Na_*x*_(H_2_O)_*y*_TaS_2_, after this, the Na_*x*_(H_2_O)_*y*_TaS_2_ was ground and added to 20 ml 0.2 g/ml aqueous glucose solution. The mixing solution was stirred for 5 min to disperse in the air and then bubbled with nitrogen to expel the oxygen for avoiding the oxidation. Na_*x*_(C_6_H_12_O_6_)_*y*_TaS_2_ can be separated from the reaction solution after about 2 h. The above dispersion was sealed and kept stirring for 6 h. Then, the solution was centrifuged at 12000 rpm for 30 min to obtain the product (marked as sample A) for further characterization. For contrast, part of the solution was standing for 1 h and the supernatant solution was centrifuged (marked as sample B). The water bubbled with nitrogen for more than 30 min (anaerobic water) could be utilized to wash off glucose and then alcohol was used to wash the sample to move water.

X-ray diffraction (XRD) patterns were recorded on a Philips X’pert Pro Super diffractometer with Cu K*α* radiation at room temperature. XRD pattern of Na_*x*_(NH_3_)_*y*_TaS_2_ was measured in air-free capillary tube, differently, and all the other intercalated samples were measured in air. The scanning electron microscopy (SEM) images were taken on a JEOL JSM-6700F field emission scanning electron microscope (FESEM, 20 kV). Transmission electron microscopy (TEM) images were obtained on a JEOL-2010 with an accelerating voltage of 200 kV. Atomic force microscopy (AFM, DI Innova Multimode SPM platform) was used for detecting the thickness. Microscopic structure investigations were carried via high-angle annular dark field (HAADF) imaging mode in an advanced JEM-ARM 200F aberration-corrected high-resolution TEM facility.

## Results and Discussion

**Fig. 1:**
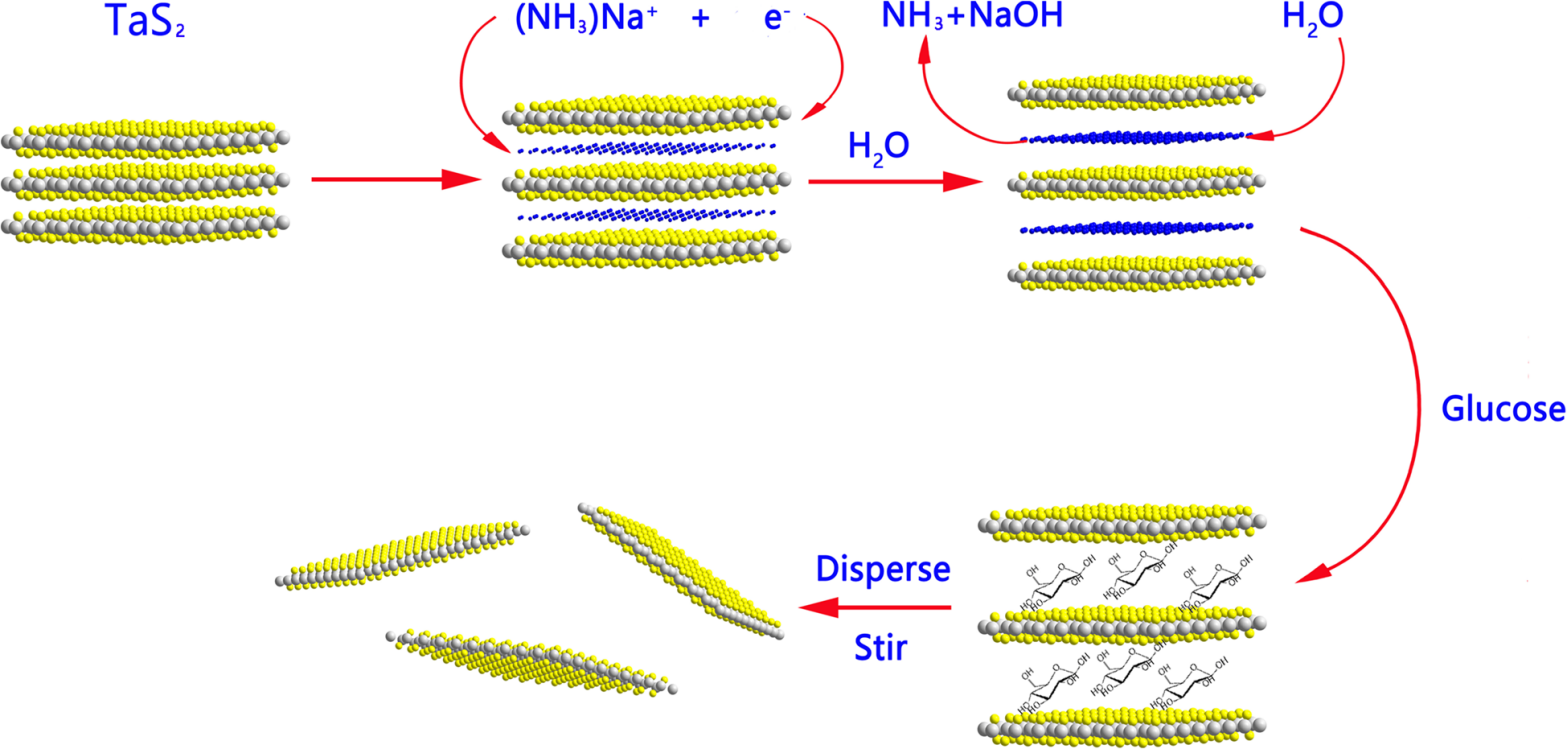
Multi-step intercalation reaction from bulk 1T-TaS_2_ to exfoliate TaS_2_ sheets

Figure 1 shows the schematic diagram of the multi-step intercalation reactions. The alkali metal-ammonia has reducibility and can be introduced simultaneously into the 1T-TaS_2_ interlayer gallery to obtain Na_*x*_(NH_3_)_*y*_TaS_2_ within a short time. Na_*x*_(NH_3_)_*y*_TaS_2_ is not stable and easily transfers to Na_*x*_(H_2_O)_*y*_TaS_2_ in water, along with the de-intercalation of Na^+^ and the generation of hydrogen [33, 34]. After transferring Na_*x*_(H_2_O)_*y*_TaS_2_ into the aqueous glucose solution, Na_*x*_(C_6_H_12_O_6_)_*y*_TaS_2_ can form and be separated after 2 h. Then, after a total of 6 h, exfoliated TaS_2_ forms eventually owing to the constant de-intercalation of Na^+^ and the weak interaction between glucose molecule and TaS_2_ layer. The flame reaction of reaction solution further confirms the de-intercalation of Na^+^, and the reaction formulas are shown (Additional file 1: Figure S1).

It is found that full dispersal of Na_*x*_(H_2_O)_*y*_TaS_2_ in glucose solution is a key factor to exfoliate TaS_2_. Additional file 1: Figure S2 shows the disperse status of Na_*x*_(H_2_O)_*y*_TaS_2_ in aerobic water, i.e., operating in air, and anaerobic water, i.e., dispelling the oxygen of water, respectively. If oxygen is bubbled before adding Na_*x*_(H_2_O)_*y*_TaS_2_, it will never be dispersed in aqueous glucose solution and exfoliated TaS_2_ cannot be obtained. In contrary, Na_*x*_(H_2_O)_*y*_TaS_2_ can be fully dispersed in aerobic water, which may be due to the hydroxylation process of the layer edge [17]. Then, the full dispersal leads to the effectively exfoliation. All products can be centrifuged and used for the characterization.

**Fig. 2:**
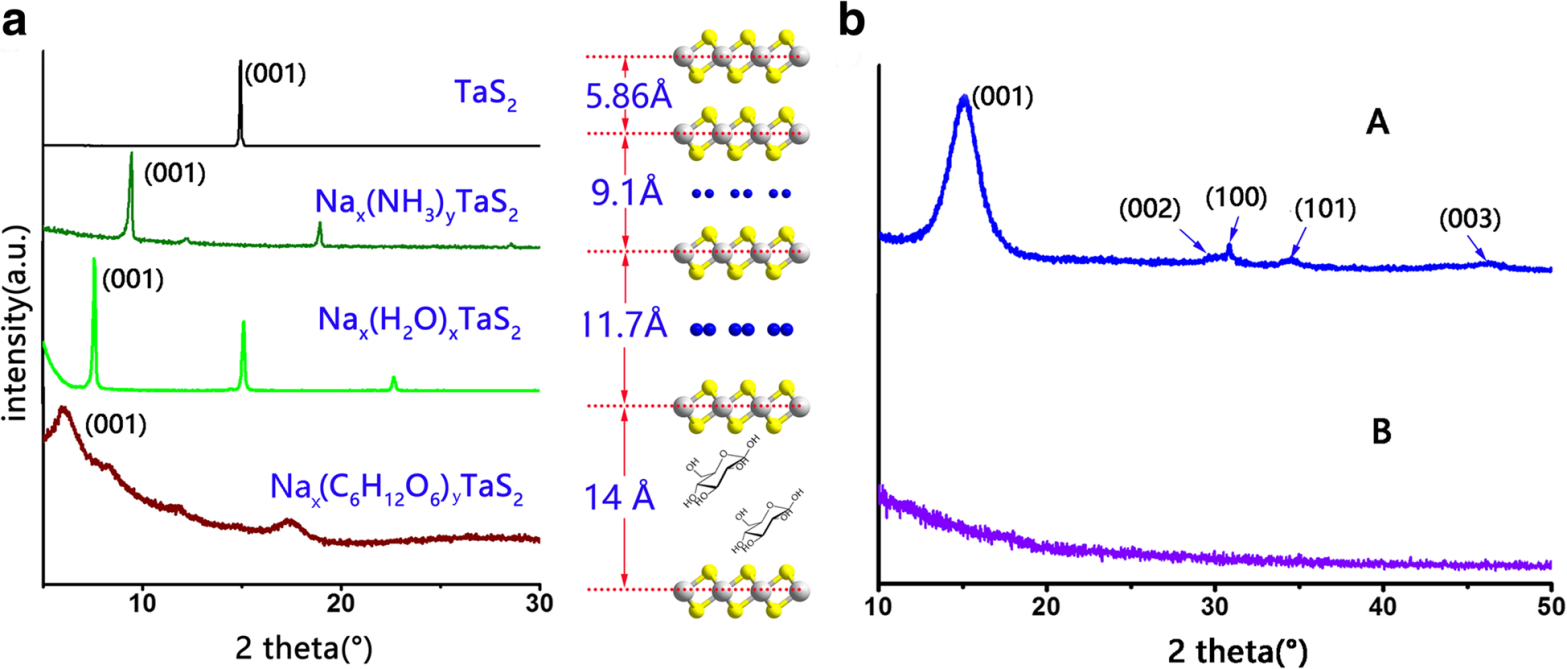
**a** XRD pattern and schematic of pristine TaS_2_, Na(NH_3_)-intercalated TaS_2_, Na(H_2_O)-intercalated TaS_2_, and C_6_H_12_O_6_-intercalated TaS_2_. **b** XRD pattern of few-layer TaS_2_(A) and monolayer TaS_2_(B)

XRD patterns (Fig. 2a) show the interlayer spacing changes of each stage. The 001 peak of pristine TaS_2_ is shifted completely toward lower angles, indicating expansion of the lattice along the *c*-axis. The interlayer spacing of Na_*x*_(NH_3_)_*y*_TaS_2_, Na_*x*_(H_2_O)_*y*_TaS_2_, and Na_*x*_(C_6_H_12_O_6_)_*y*_TaS_2_ are 9.1, 11.7, and 14 Å, respectively. Comparing with the 5.86 Å of TaS_2_, the interlayer spacing of Na_*x*_(C_6_H_12_O_6_)_*y*_TaS_2_ has increased by 8.1 Å, which would greatly weaken the Van der Waals’ force of interlayer and is of advantage to the exfoliation of TaS_2_.

Figure 2b (A) shows the XRD pattern of sample A. The 001, 002, 100, 101, and 003 peaks are completely in accordance with 1T-TaS_2_. The sharp 100 peak, and wide and strong 00 *l* reflections indicate the *ab* extensional orientation of the nanosheets. The average thickness is estimated to be 3 nm by Scherrer equation based on the FWHM of 001 peak. Besides, The XRD pattern (Fig. 2b (B)) of sample B shows that the 001 peak vanishes completely, i.e., the period of *c*-axis disappears, meaning the formation TaS_2_ monolayers. Besides, as the same structure, 1T-TiS_2_ can also be exfoliated by our method. The XRD pattern of exfoliated TiS_2_ is shown in Additional file 1: Figure S3. The average thickness of exfoliated TiS_2_ is estimated to be 4 nm by Scherrer equation. This shows the potential of our method to expand into other TMDs.

Additional file 1: Figure S4 shows the Raman spectra of bulk TaS_2_ and exfoliated nanosheets. There are two Raman features that can be determined, which are E_1g_ and A_1g_ modes [35, 36]. From bulk to nanosheet, the E_1g_ and A_1g_ modes show some shifts. These shifts are probably attributed to the decrease of the force constant resulted from the weakening of the interlayer Van der Waals force between layers. There is also a new mode of vibration which may come from structure changes. The FWHM of exfoliated TaS_2_ is larger than that of bulk, which also indicates the decrease of thickness

**Fig. 3:**
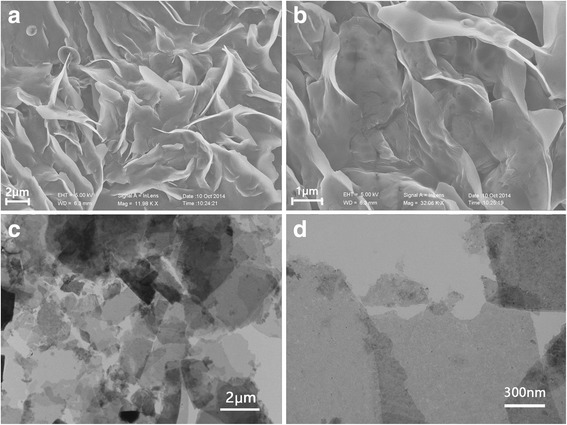
Morphology, thickness distribution of exfoliated TaS_2_ thin film. **a**, **b** Typical SEM images show the flower-like morphology with several micrometers in size. **c**, **d** Typical TEM images show the morphology of synthetic samples and the micron-sized proportion

The FESEM images in Fig. 3a, b clearly reveal flake-like morphology with several micrometers in size. One remarkable result is the case that most of the TaS_2_ sheets have micro-sized proportion. TEM images of Fig. 3c, d further show the morphology of the sample.

**Fig. 4:**
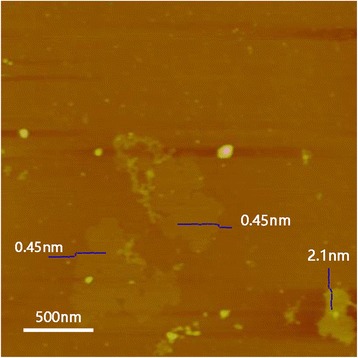
AFM image of exfoliated TaS_2_ nanosheets show the thickness of monolayer and three to four layers of TaS_2_ sheets are about 0.45 and 2.1 nm, respectively

Figure 4 shows the AFM analysis performed on samples which are deposited on muscovite substrate. We measured three sheets (0.45, 0.45, and 2.1 nm, respectively). The thickness of 0.45 nm is within the permissible range of the 001 interlayer spacing of 5.86 Å, and the polyhedron size of 2.93 Å formed by Ta and S atoms (more evidence can be seen in HADDF image of Additional file 1: Figure S5), which is the direct evidence of the formation of monolayer TaS_2_. Meanwhile, the 2.1-nm-thick nanosheet stands for three to four layers of TaS_2_.

Our approach can produce micro-sized sheets (maximum lateral size is 5 μm) and monolayer TaS_2_ (minimum thickness is 0.45 nm). Ultrasound is not necessary and may suffer the size of the exfoliated nanoflakes. More details about the differences between different exfoliation techniques are shown in Additional file 1: Table S1.

**Fig. 5:**
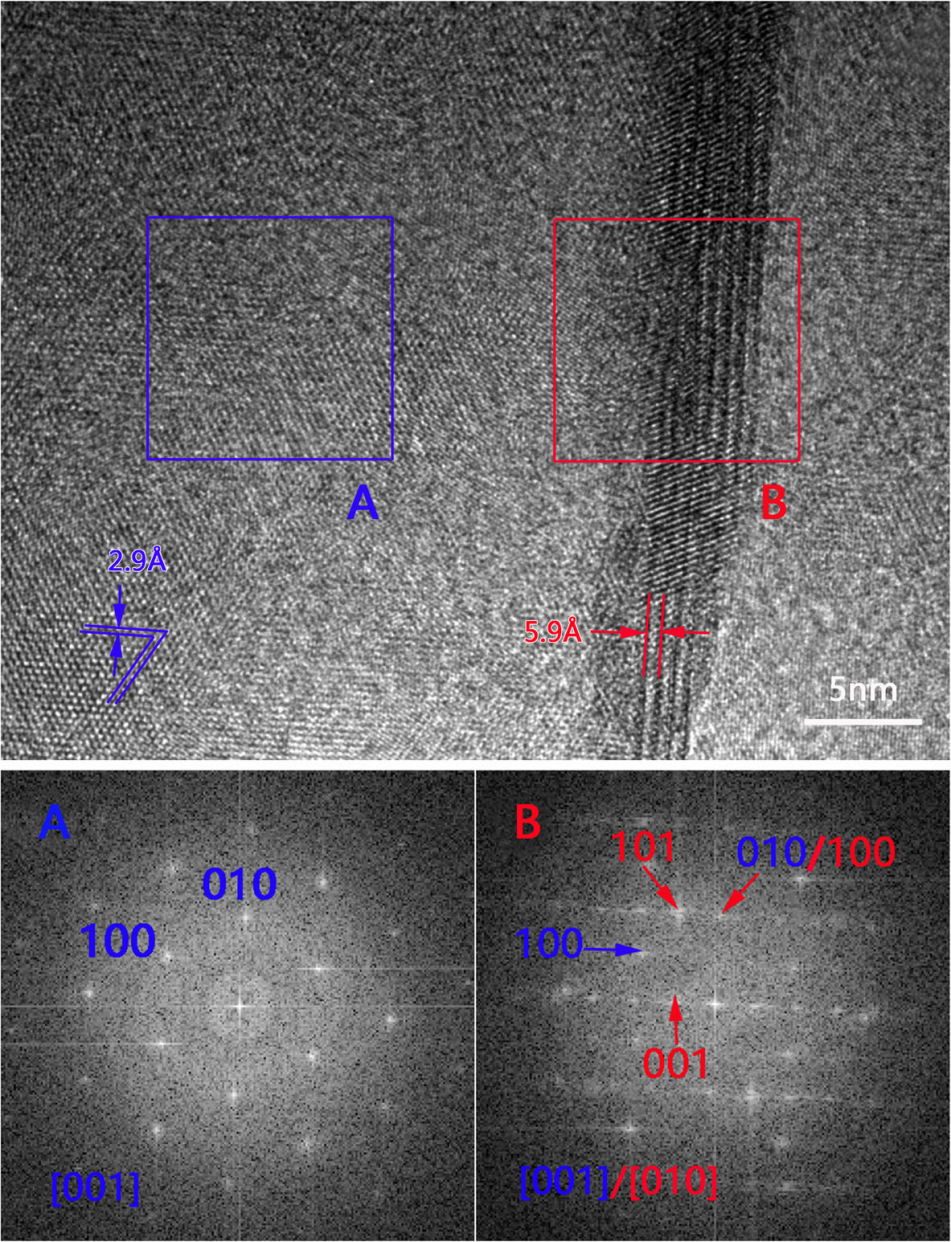
Characterization of few-layer TaS_2_. HRTEM image of crisscross structure and FFT pattern of A and B regions. A is predictable [001] zone axis TaS_2_ nanosheet, while B is concurrence of [001]/[010] zone axis. Among this, 101 and 001 spots of 1T-TaS_2_ are identified

Figure 5 displays the high-resolution transmission electron microscopy (HRTEM) image and fast Fourier transformation (FFT) patterns of a crisscross structure. The lattice fringes of 2.9 and 5.9 Å are consistent with 100 and 001 planes of bulk TaS_2_, respectively. FFT patterns were carried on the typical [001] zone axis (A) and [001]/[010] zone axis (B), respectively. In addition to the overlapping part, 101 and 001 diffraction spots of 1T-TaS_2_ on B region are identified, indicating that the few-layer nanosheets adopt 1T-TaS_2_ structure.

**Fig. 6:**
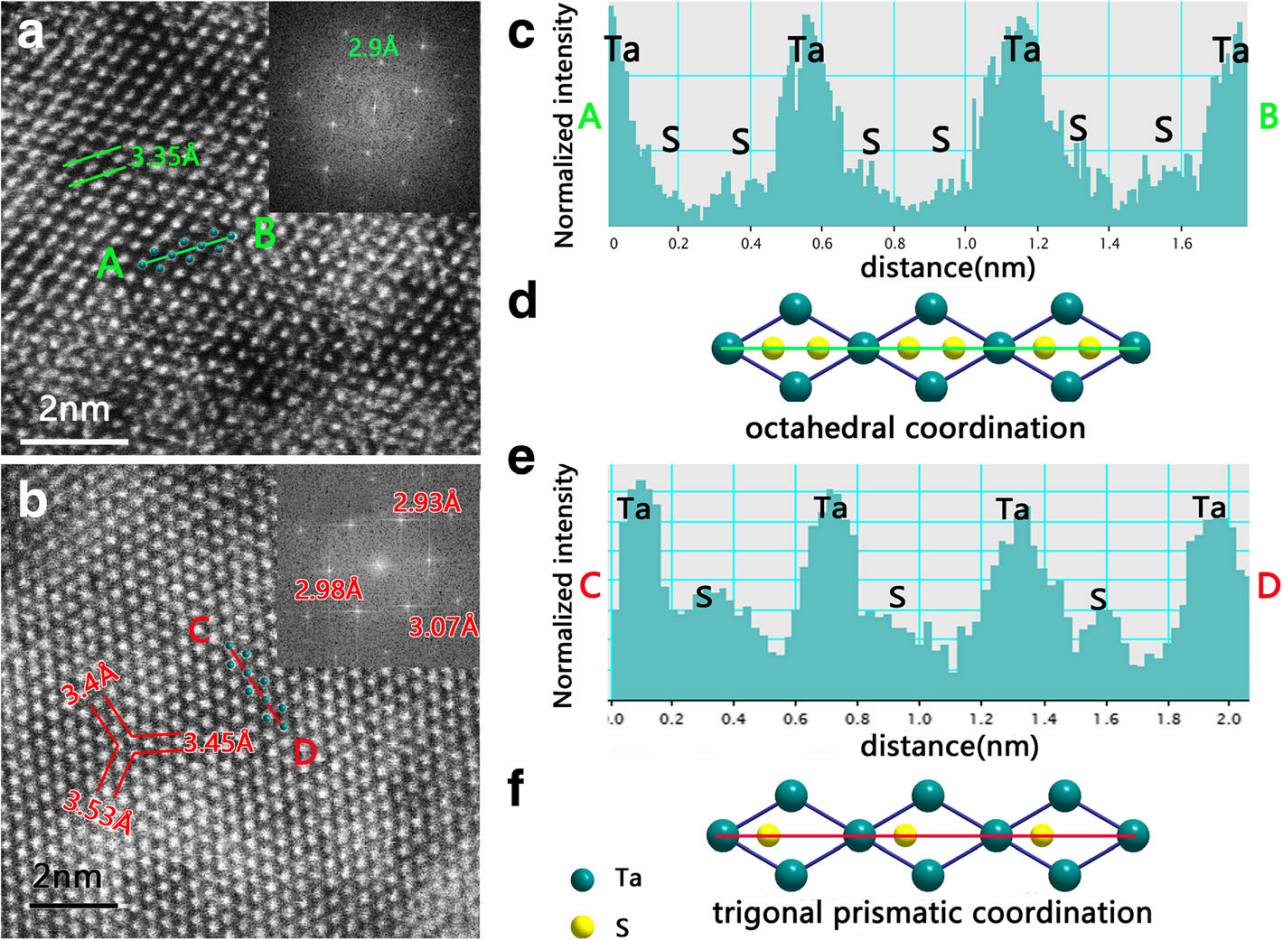
Atomic structural analysis of monolayer TaS_2_. **a** Typical HADDF-STEM image of TaS_2_ shows clear atomic patterns of octahedral TaS_2_ and its FFT pattern. **b** A typical HADDF-STEM image of TaS_2_ shows clear atomic patterns in monolayer triangular TaS_2_ and its distorted FFT pattern. **c** Bottom intensity profiles of 1T-TaS_2_ along AB line, and the structural model of octahedral TaS_2_ are showed for contrast. **d** Bottom intensity profiles along CD line exhibit the location of Ta and S atoms which indicate atomic position of the trigonal prismatic and the structural model of trigonal TaS_2_

We also carried out atomic structure investigations via HAADF imaging mode in an advanced aberration-corrected high-resolution STEM facility. Figure 6a recorded at a few-layer TaS_2_ sheet shows a 1T atomic structure which can be clearly seen in the HAADF image. The distance between two Ta atoms is 3.35 Å, and the FFT pattern shows the normal 2.9 Å spot of 100. To further explore the structure, we have utilized the Z-contrast in HAADF-STEM images (Fig. 6a). The contrasts contributed by S atoms distribute symmetrically along the AB line (Fig. 6c). The results are consistent with the octahedral coordination of S-Ta-S in 1T-TaS_2_ structure (Additional file 1: Figure S6a) [37]. S atoms are symmetrically distributed around the Ta sites (Fig. 6c).

When we further characterized the monolayer nanosheet, an interesting phenomenon is found that the atomic distance between two Ta atoms from three directions are 3.4, 3.45, and 3.53 Å, respectively (Fig. 6b). The corresponding FFT pattern shows a distorted hexagon (2.93, 2.98, and 3.07 Å, respectively) which indicates the lattice distort in *ab* plane (Fig. 6b). The intensity profile recorded along CD line (Fig. 6e) differs from the case of few-layer TaS_2_ (Fig. 6c). The contrasts contributed by S atoms distribute asymmetrically along the CD line, and its relative intensities are much stronger than that in Fig. 6c, which means another S-Ta-S coordination in the monolayer structure. Note that there are two S-Ta-S coordinated situations in TaS_2_, the results are consistent with the 1H single layer structure with trigonal prism coordination of S-Ta-S (Additional file 1: Figure S6b) [38]. In this case, the S atoms are asymmetrically distributed around Ta atom along the CD line, and the overlap of two S atoms along *c*-axis (the electron beam direction) result in the enhancement of the signal from the S atoms (Fig. 6f). Therefore, 1H structure with trigonal prism coordination of S-Ta-S in monolayer TaS_2_ sheet is proposed. Such a single layer structure may be unstable, and the lattice distortion would probably occur. This structure may be responsible for the new vibration mode in Raman result. Of course, the essence of lattice distortion in monolayer TaS_2_ should be further explored. Meanwhile, different structural phases of TaS_2_ sheets may be exploited for different device applications.

## Conclusions

In summary, we have developed an efficient exfoliation method for the exfoliation of tantalum disulfide and obtained micron-sized monolayer to few-layer sheet. This is a pure chemical exfoliation method in which ultrasound is not necessary. Monolayer and few-layer sample have all been characterized in detail, and coordination change from octahedron to triangular prism is found on monolayer TaS_2_. This really warns everyone to pay attention to the point of property variation caused by structural changes in exploring the finite-size effect, while few-layer TaS_2_ still keeps the 1T structure.

Briefly, the present work not only provides an efficient ultrasound-free strategy for synthesizing high-exfoliated functional nanosheets but also explores the structure of monolayer and few-layer TaS_2_. The present work will provide insights to better understanding 2D materials and their physical and chemical properties, and offer more opportunities for their applications.

## Additional file

Additional file 1: Supporting information. (DOCX 1440 kb)
