# Supplementary material for: Toward Exploring the Structure of Monolayer to Few-layer TaS2 by Efficient Ultrasound-free Exfoliation
Source: Nanoscale Res Lett. 2018 Jan 15;13:20. doi: 10.1186/s11671-018-2439-z (PMC5768583; doi:10.1186/s11671-018-2439-z)
Supplement: Additional file 1: — Supporting information. (DOCX 1440 kb) [file 11671_2018_2439_MOESM1_ESM.docx]

**Supporting Information**

Toward Exploring the Structure of Monolayer to Few-layer 1T TaS_2_ by Efficient Ultrasound-free Exfoliation

Yiwei Hu, Qiaoyan Hao, Baichuan Zhu, Biao Li, Zhan Gao, Yan Wang and Kaibin Tang^*^

Division of Nanomaterials and Chemistry, Hefei National Laboratory for Physical Sciences at the Microscale, Department of Chemistry, University of Science and Technology of China, Hefei 230026, P.R. China. E-mail: kbtang@ustc.edu.cn; Fax: +86-551-63606266; Tel: +86-551-63601791

| Work | Maximum lateral size | Minimum thickness | Use of ultrasound | Object |
| --- | --- | --- | --- | --- |
| This work | 5 μm | 0.45nm | × | TaS_2_ |
| Ref. 7 | 0.5 μm | 1 nm | √ | WS_2_ |
| Ref. 13 | 0.2 μm |  | √ | MoS_2_ |
| Ref. 18 | 3 μm |  | √ | MoS_2_ |
| Ref. 19 | 3 μm | 1.97nm | √ | TiS_2_ |
| Ref. 21 | 0.5 μm | 1 nm | √ | MoS_2_ |
| Ref. 25 | 0.5 μm | 3 nm | √ | MS_2_(M=Mo,W,Ta,Ti) |
| Ref. 27 | 0.3 μm | 3.5 nm | √ | MoS_2_ |
| Ref. 35 | 20 μm | 1 nm | √ | MS_2_(M=Mo,W,Ta,Ti) |

**Table S1** Contrasts of different exfoliation techniques

Using the maximum lateral size (found from TEM, SEM or AFM images in references) and minimum thickness (found from AFM images) as judgment, we contrast different exfoliation techniques.


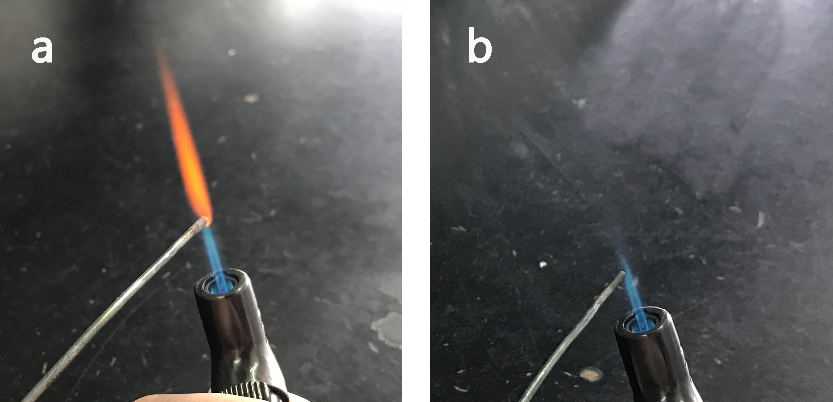


**Fig. S1**. a) The flame reaction of reaction solution indicates the de-intercalation of Na^+^.

b) Glucose solution for contrast.

The reaction formulas are as follows:

TaS_2_ + Na + NH_3_ → Na_x_(NH_3_)_y_TaS_2_

Na_x_(NH_3_)_y_TaS_2_ + H_2_O → Na_a_(H_2_O)_b_TaS_2_ + NaOH + NH_3_ + H_2_

Na_a_(H_2_O)_b_TaS_2_ + C_6_H_12_O_6_ → Na_d_(C_6_H_12_O_6_)_e_TaS_2_ + H_2_O + NaOH + H_2_

Na_d_(C_6_H_12_O_6_)_e_TaS_2_ + H_2_O → TaS_2_ + C_6_H_12_O_6_ + NaOH + H_2_


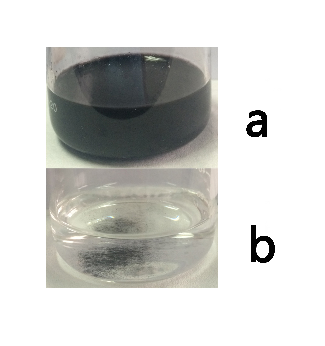


**Fig. S2**. The disperse status of Na_x_(H_2_O)_y_TaS_2_ in aerobic water (a) and anaerobic water (b)


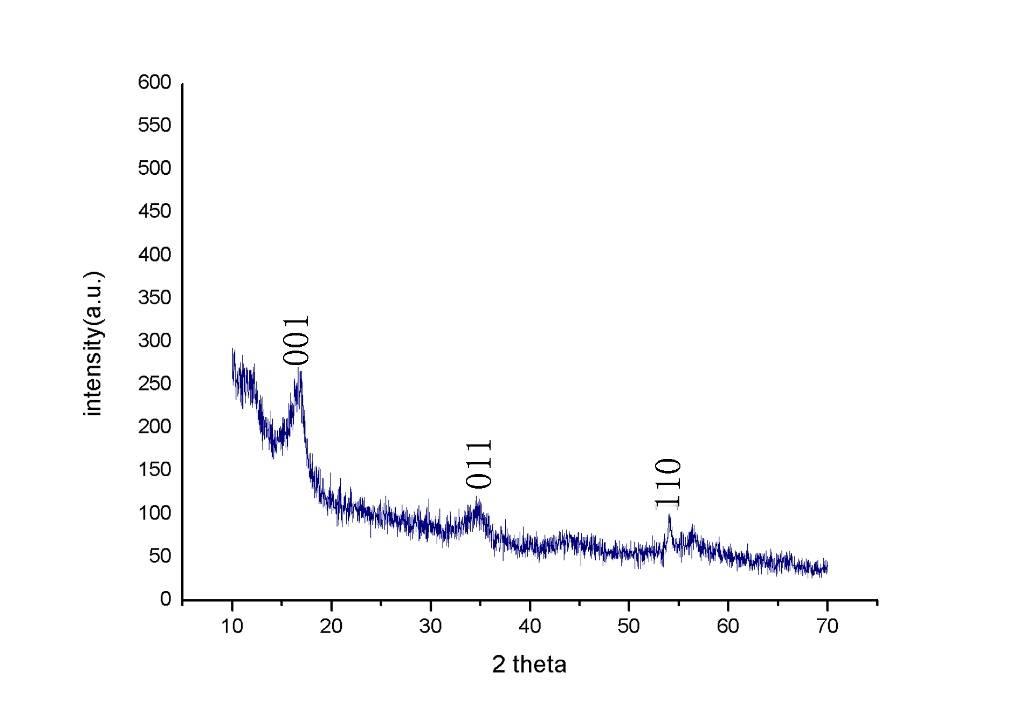


**Fig. S3**. XRD pattern of exfoliated TiS_2_


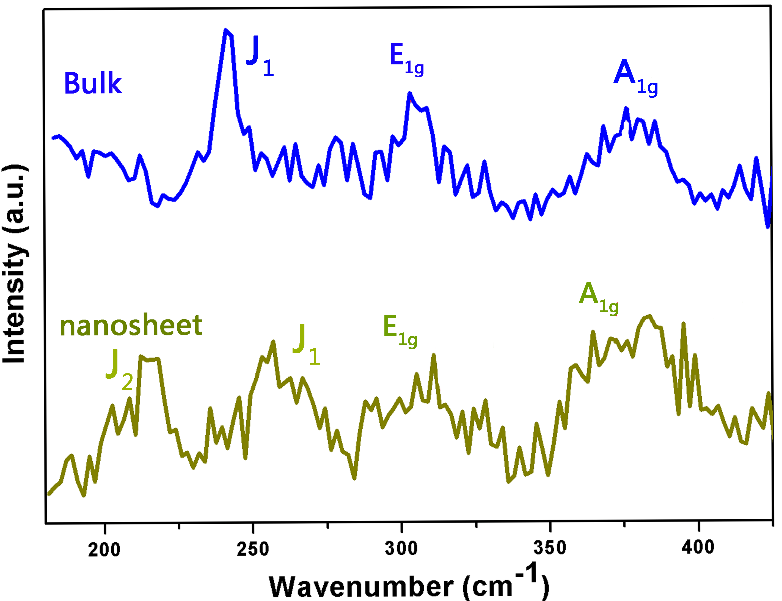


**Fig. S4** Raman spectra of bulk TaS_2_ and exfoliated nanosheet.

There are two Raman features that can be determined which are E_1g_ and A_1g_ modes. From bulk to nanosheet, the J_1_, E_1g_ the A_1g_ modes show some shift. The new Raman feature J_2_ in the spectra of exfoliated nanosheet probably comes from the 1H structure what we’ll talk about later.


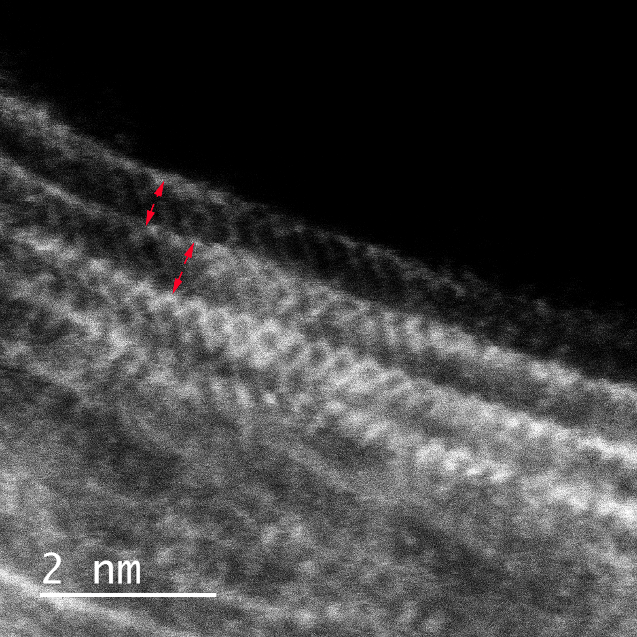


**Fig. S5.** HADDF imaging mode of cross section in an advanced aberration-corrected high resolution STEM facility.

We can clearly see the space of interlayer from Fig. S4. Regardless of the atomic radius, the thickness of monolayer TaS_2_ is only 0.29nm according to the location of atoms. However, it should be thicker than 0.29nm because of the atomic radius. Owing to the Van der Waals’ force, the interlayer spacing is not filled. So, the spacing should be thinner than the interlayer spacing 0.59nm (001). This proves the AFM result from another perspective. Besides, Ref. 35 shows that the thickness of MoS_2_, WS_2_ and TaS_2_ are 1nm, 1nm and 0.9nm, respectively. These are also thinner than the theoretical 1.23nm, 1.24nm and 1.21nm thickness of two layers (or one period for 2H structure). Besides, the AFM image of the reference (Wenwen Zhao, Bohua Dong, Zenglong Guo, Ge Su, Rongjie Gao, Wei wang and Lixin Cao, *ChemComm*, 2016, 52, 9228-9231) shows that the thickness of monolayer VSe_2_ is 0.4nm. This is thinner than its theoretical interlayer spacing of 0.61nm.


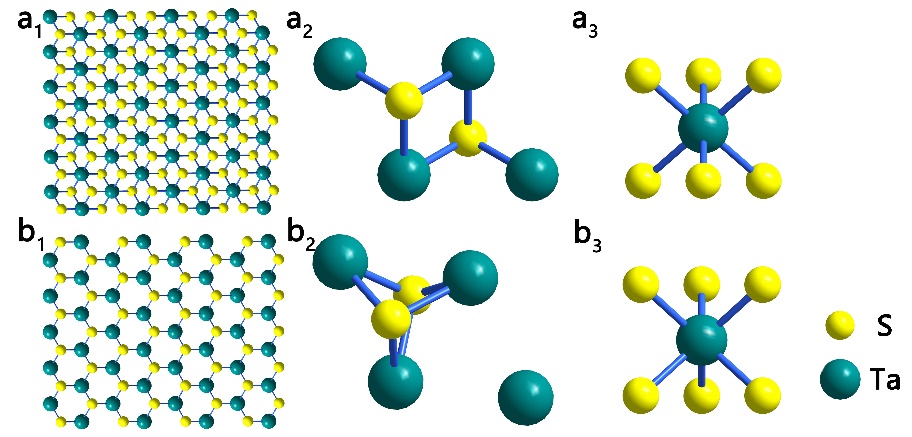


**Fig. S6**. a1), b1) The corresponding simulated structural model of octahedral coordination (1T) and triangular prism coordination (2H). a2), b2) The relative location of Atoms in one cell. a3), b3) Coordination mode of Ta atom.

Fig. S6 shows the structural model, the 3D diagram and the coordination mode of the corresponding structure. Fig. S6(a1) is the planar graph of 1T structure. It has a tetragonal symmetry (D_3d_) and corresponds to an octahedral coordination of Ta atoms (Fig. S6(a3)). One of sulfur layers is shifted compared to the other resulting in an AbC stacking sequence (Fig. S6 (a2)). There are two S atoms along the long-diagonal of the rhomboid formed by Ta atoms. One is above the Ta plane and the other is beneath it. So, from the c direction, we can see the symmetrical intensity of two S atoms (Fig. 6c). Fig. S6(a2) is the 3D diagram of one 1T-TaS_2_ cell. We can clearly see from it that one S atom is above and the other is underneath. Fig. S6(a3) shows the octahedral coordination mode of 1T structure. In contrast, the trigonal prismatic phase is also referred to as the 2H phase (or 1H in the case of a single layer) [6] and can be described by hexagonal symmetry (the D_3h_ group) and corresponds to a trigonal prismatic coordination of the Ta atoms (Fig. S6(b3)). This geometry means that in single layers, the sulfur atoms are vertically aligned along the c-axis and the stacking sequence is then AbA where A and b denote S and Ta atoms, respectively (Fig. S6(b2)). There are also two S atoms in one rhomboid, but the two S atoms are overlapped. So, from the c direction, we can only see intensity of one S atom (Fig. 6d). Fig. S6(b2) is the 3D diagram of one 1H TaS_2_ cell and Fig. S6(b3) is the triangular prismatic coordination of 1H TaS_2_.
